# Analgesic and Anti-Inflammatory Activities of Sophocarpine from *Sophora viciifolia* Hance

**DOI:** 10.1155/2021/8893563

**Published:** 2021-11-08

**Authors:** F. L. Wang, H. Wang, J. H. Wang, D. X. Wang, Y. Gao, B. Yang, H. J. Yang, Y. B. Ji, G. S. Xin

**Affiliations:** ^1^School of Pharmacy, Engineering Research Center for Medicine, Harbin University of Commerce, Harbin 150076, China; ^2^Engineering Research Center of Natural Anticancer Drugs, Ministry of Education, Harbin 150076, China; ^3^Key Laboratory for Drug Research on Prevention and Treatment of Geriatric Diseases of Heilongjiang Province, Harbin 150076, China

## Abstract

*Sophora viciifolia* Hance is an edible plant used in traditional Chinese medicine. Sophocarpine, a tetracyclic quinolizidine alkaloid, is one of the most abundant active ingredients in *Sophora viciifolia* Hance. Here, we study the analgesic and anti-inflammatory effects, as well as the acute toxicity of sophocarpine from *Sophora viciifolia* Hance in mice. Sophocarpine (20, 40, and 80 mg/kgbw) significantly prolonged the delay period before a hot plate reaction occurred (all *P* < 0.05), and the delay before a tail-flick response was induced by a warm bath (*P* < 0.05; *P* < 0.01). Sophocarpine (40, 80 mg/kg) resulted in dose-dependent inhibition of the writhing reaction induced by acetic acid in mice (*P* < 0.05; *P* < 0.001, respectively). Sophocarpine (80 mg/kg) reduced the total duration of a formalin-induced pain response (*P* < 0.05). Sophocarpine prolonged the foot-licking latency of mice after the hot plate reaction, and this effect was antagonized by calcium chloride and enhanced by verapamil. Sophocarpine (20, 40, and 80 mg/kg) significantly inhibited xylene-induced ear edema (*P* < 0.01; *P* < 0.001; *P* < 0.001, respectively) and the penetration of acetic acid-induced dye into the peritoneal cavity (*P* < 0.01; *P* < 0.01; *P* < 0.001, respectively). It also reduced the levels of proinflammatory cytokine interleukin (IL)-1*β*, IL-6, and prostaglandin E2 (*P* < 0.05, *P* < 0.01, *P* < 0.001) and those of serum nitric oxide (*P* < 0.05). The results of this study suggest that sophocarpine possesses certain analgesic and anti-inflammatory activities, which may be related to calcium and inhibition of the secretion of inflammatory factors.

## 1. Introduction

Pain is usually related to inflammation and is the most common manifestation of many diseases [1]. It is associated with the pathophysiology of various clinical conditions, such as arthritis, cancer, and vascular diseases [2]. In inflammatory pain conditions, proinflammatory cytokines (interleukin 1 [IL-1], IL-6, tumor necrosis factor alpha [TNF-*α*]) and various small molecules (adenosine triphosphate, bradykinins, prostaglandins) are recruited to the site of injury and act on nociceptors, leading to hyperalgesia, allodynia, and spontaneous pain [3]. High-output nitric oxide (NO) generated by inducible NO synthase can lead to deleterious consequences, such as septic shock and inflammatory diseases [4]. Conventional analgesic and anti-inflammatory agents are very often misused. These therapies occasionally fail or produce partial responses. In addition, serious side effects (such as nephrotoxicity, hepatotoxicity, and gastric ulceration) and low efficacy limit the use of these agents [5]. The analgesic and anti-inflammatory effects of natural medicine have become the focus of attention. *Sophora viciifolia* Hance, which belongs to the butterfly flower family, grows at an altitude of 1,300–2,500 m and is known as Baicihua in China. It is mainly distributed in the Yunnan, Guizhou, Sichuan, and Ningxia provinces. Conventionally, the flower of *Sophora viciifolia* Hance is used as a remedy for night sweats, heat stroke, and edema, among other conditions [6]. It is thought that consumption of the flower in springtime may eliminate the “toxins” that have accumulated in the human body during the previous season. The active ingredients of Baicihua include sophoramine, sophordine, matrine, oxymatrine, sophocarpine (SC), and oxysophocarpine. At present, there is limited research on the anti-inflammatory and analgesic effects of SC. The purpose of the present study was to investigate the possible analgesic and anti-inflammatory activities of SC in animal models.

## 2. Materials and Methods

### 2.1. Plant Materials

Flowers of *Sophora viciifolia* Hance were collected in the Yun-Nan province of China in March 2015 and identified by Professor B. Yang (School of Pharmacy, Harbin University of Commerce, Harbin, China). SC was isolated from *Sophora viciifolia* Hance [7] with a purity of >98%, as determined by a Waters HPLC system equipped with a UV detector and C18 column (250 × 4.6 mm; 5 *μ*m). The analyses were performed with a column at 25°C. The flow rate was maintained at a constant 1.0 mL/min. A volume:volume ratio of 12 : 88 of acetonitrile: 0.05 mol/L KH_2_PO_4_ and 2.0 mL/L triethylamine was used as the mobile phase. The detection wavelength was 205 nm. The quantitative determination of SC was performed using an external standard based on the peak area. A reference compound with a purity of >98% was obtained from Shanghai Aladdin Biochemical (Shanghai, China).

### 2.2. Chemicals and Reagents

The following reagents were used in this study: aspirin (Shineway Pharmaceutical; Shijiazhuang, China), normal saline (Sanlian Pharmaceutical; Harbin, China), acetic acid and formalin 37% (Sinopharm Chemical Reagent Beijing; Beijing, China), verapamil (Central Pharmaceutical; Tianjin, China), xylene (Jingdongtianzheng Precision Chemical Reagent Factory; Tianjin, China), dexamethasone sodium phosphate injection (Zhuofeng Pharmaceutical; Zhengzhou, China), and carrageenan (Sigma–Aldrich; St. Louis, MO, USA). The NO Assay Kit was obtained from Beyotime (Shanghai, China). The prostaglandin E2 (PGE2), IL-1*β*, and IL-6 enzyme-linked immunosorbent assay (ELISA) kits were obtained from Nanjing Jiancheng Bioengineering Institute (Nanjing, China). All other chemicals used in the experiments were of analytical grade.

### 2.3. Animals

Kunming mice (males, weight: 18–22 g; SCXK(Ji)2016-0003) were provided by Changchun Yisi Experimental Animal Technology. Animals were housed in polycarbonate cages in a temperature- and humidity-controlled environment with a 12-h light/dark cycle and free access to food and water. All animal experiments complied with the National Institutes of Health (Bethesda, MD, USA) Guide for the Care and Use of Laboratory Animals (NIH Publications No. 8023, revised 1978).

### 2.4. Acute Toxicity Tests

The animals were fasted overnight prior to the administration of SC. Mice in five groups (six animals per group) were intraperitoneally (i.p.) injected with different doses of SC (mg/kg). The animals were observed for 1–2 h after administration for any acute signs of behavioural toxicity. At 48 h after treatment, the number of deaths was determined. The LD_50_ of SC (i.e., dose resulting in 50% mortality) was determined by Probit regression statistical analysis [8].

### 2.5. Animal Grouping and Dosing

**Figure 1:**
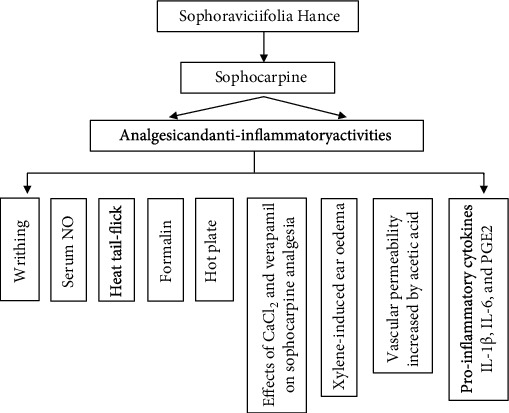
Schematic representation of the experiment. Abbreviations: CaCl_2_: calcium chloride; IL-1*β*: interleukin 1*β*; interleukin 6: IL-6; NO: nitric oxide; PGE2: prostaglandin E2.

For the analgesic and anti-inflammatory activity test, mice were randomly divided into five groups (i.e., negative control, positive control, and three test groups) comprising 10 animals each (Figure 1). Negative control animals received normal saline (10 mL/kg, i.p.), while positive control animals were treated with standard drugs (aspirin, 100 mg/kg, intragastric; dexamethasone, 2.5 mg/kg, i.p.). The remaining three groups received different doses (20, 40, and 80 mg/kg, i.p.) of SC. Access to food, but not water, was withdrawn 2 h prior to drug administration.

### 2.6. Writhing Test

At 30 min after drug administration, the mice received an i.p. injection of 1% volume/volume acetic acid solution (10 mL/kg). The mice were placed individually in transparent cages. The number of acid-induced writhes was counted for 20 min. For the purpose of scoring, a writhe was indicated by stretching of the abdomen and/or simultaneous stretching of at least one hind limb [9].

### 2.7. Measurement of NO

After the writhing test, the femoral artery was immediately cut to obtain a blood sample. The total blood sample was centrifuged at 1,097 × *g* for 10 min to separate the serum. The levels of NO in the serum were determined using the NO Assay Kit [10].

### 2.8. Heat Tail-Flick Test

The time required by mice to withdraw (flick) the tail was considered the reaction time [11]. Observations were recorded prior to and at 0.5, 1, 1.5, and 2 h after drug administration.

### 2.9. Formalin Test

Mice received a subcutaneous injection (20 *μ*L of 1% formalin prepared in 0.9% saline) into the dorsal hind paw and were immediately transferred in a transparent box for observation [12]. The duration of the reaction time (paw licking or biting) was determined from 0–5 min (first phase) and 15–30 min (second phase) after formalin injection.

### 2.10. Hot Plate Test

Mice were placed in an aluminium hot plate maintained at a temperature of 55 ± 0.5°C [11]. The response latency to a discomfort reaction (licking paws or jumping) was determined before and at 15, 30, 60, 90, and 120 min after drug administration. The cut-off time was 60 s.

### 2.11. The Effects of Calcium Chloride (CaCl_2_) and Verapamil on SC Analgesia

A total of 40 mice with a basic pain threshold within 5–30 s were randomly divided into four groups: one group treated with normal saline (i.p.) and three groups treated with 80 mg/kg SC (i.p.). After 45 min, one group was given 0.3 mg/kg verapamil intravenously (i.v.), the other group given 5 mg/kg CaCl_2_ (i.v.), and the pain threshold was measured again after 15 min [13].

### 2.12. Xylene-Induced Ear Oedema Test

The mice were treated with normal saline, SC, or dexamethasone for 4 d. On day 4, at 1 h after administration, xylene (30 *μ*L) was applied to both the anterior and posterior surfaces of the right ear [14]. The left ear was used as control. After 1 h, the mice were sacrificed, and sections (thickness: 9 mm) were obtained from both ears using a cork borer. Any increase in weight caused by the irritant was subsequently measured by subtracting the weight of the untreated left ear section from that of the treated right ear section.

### 2.13. Acetic Acid-Induced Vascular Permeability Test

At 1 h after administration, mice received an injection of 1% Evan's blue solution (5 mL/kg, i.v.). After 5 min, each mouse received an injection (i.p.) of 1% acetic acid solution (10 mL/kg). Twenty minutes later, the mice were sacrificed, and the concentration of Evan's blue in the fluid of the peritoneal cavity was determined by measuring the absorbance at 590 nm [15].

### 2.14. Analysis of IL-1*β*, IL-6, and PGE2

At 1 h after drug administration, paw edema was induced by subcutaneously injecting 20 *μ*L of freshly prepared 1% carrageenan solution into the plantar surface of the right hind paw [16]. The mice were sacrificed after 4 h, and the toes of the right hind paw were homogenized and diluted to form a 10%homogenate. This was centrifuged at 4:C and 9,391 × *g* for 15 min, and the supernatant was collected. The levels of IL-1*β*, IL-6, and PGE2 were determined using ELISA kits according to the instructions provided by the manufacturer [17].

### 2.15. Statistical Analysis

All experimental data are represented as the mean ± standard deviation. Analysis of variance was performed using the statistical software SPSS version 18.0 (IBM, Armonk, NY, USA). *P* < 0.05 was assumed to indicate a statistically significant difference.

## 3. Results and Conclusions

### 3.1. Acute Toxicity Test

The administration of various doses of SC (i.p.) up to 80 mg/kg did not cause an increase in lethality. The LD_50_ value of SC was 115 mg/kg, the LD_1_ value was 85 mg/kg, and a dose not exceeding LD_1_ was selected as the highest dose.

### 3.2. Acetic Acid-Induced Writhing Test

The administration of SC (40, 80 mg/kg) caused significant inhibition (*P* < 0.05; *P* < 0.001, respectively) of the nociception induced by acetic acid. At a dose of 80 mg/kg, SC produced a maximum protection of 47.7%. The results were comparable to those obtained for the standard drug aspirin (78.6% inhibition at 100 mg/kg; Table 1).

### 3.3. Levels of Serum NO

**Figure 2:**
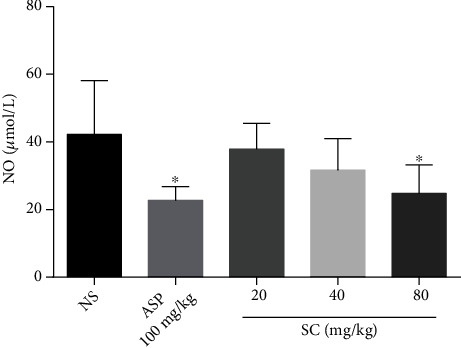
Effect of SC on the serum levels of NO in mice. Abbreviations: ASP: aspirin; SD: standard deviation; NO: nitric oxide; NS: normal saline; SC: sophocarpine. Values are expressed as mean ± SD (*n* = 10). ^∗^*P* < 0.05, compared with the NS control group.

There was a significant decrease (*P* < 0.05) in NO levels in the group treated with aspirin (100 mg/kg) compared with the control group. Similarly, treatment with SC (80 mg/kg) markedly decreased (*P* < 0.05) the levels of NO (Figure 2). The relationship between analgesia and NO in brain tissue needs further experimental study.

### 3.4. Heat Tail-Flick Response

Compared with NS, treatment with SC (20, 40, and 80 mg/kg) significantly prolonged the delay before a tail-flick was generated in response to heat (*P* < 0.05; *P* < 0.01). The analgesic effect increased in parallel with the dose, and the efficacy lasted for 2 h (Table 2).

### 3.5. Formalin Test

**Figure 3:**
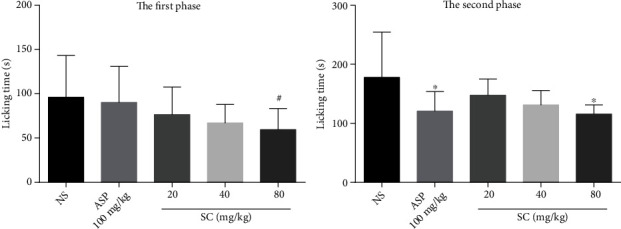
Effect of SC in the formalin test. Abbreviations: ASP: aspirin; NS: normal saline; SD: standard deviation; SC: sophocarpine. Values are expressed as mean ± SD (*n* = 10). ^#^*P* < 0.05, compared with the NS control group (first phase); ^∗^*P* < 0.05, compared with the NS control group (second phase).

Treatment with SC (80 mg/kg) caused significant (*P* < 0.05) inhibition in the second phase of formalin-induced pain. The paw licking or biting time of mice was 177.9 ± 76.70 s in the NS control group and 115.7 ± 15.72 s in the 80 mg/kg SC group. A maximum inhibition of 34.96% was induced by 80 mg/kg SC in the second phase of the formalin test. Similarly, treatment with aspirin suppressed the duration of the reaction of the animals in the second phase (Figure 3).

### 3.6. Hot Plate Test

Treatment with three doses of SC (20, 40, and 80 mg/kgbw) significantly increased the latency time in a dose-dependent manner (all *P* < 0.05). The highest inhibition of response latency was exhibited at the higher dose of 80 mg/kg SC (17.64-34.37%), and the maximum effect of SC was observed at 60 min after drug administration (Table 3).

### 3.7. Effects of CaCl_2_ and Verapamil on SC Analgesia

**Figure 4:**
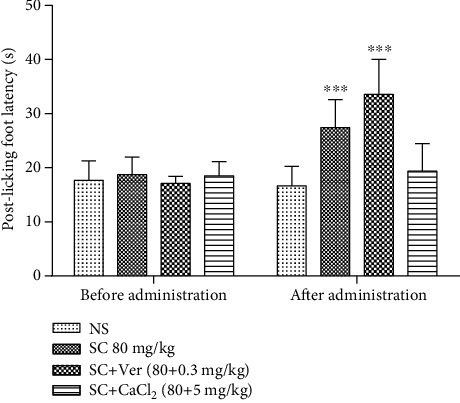
Effects of CaCl_2_ and verapamil on analgesia caused by SC. Abbreviations: CaCl_2_: calcium chloride; NS: normal saline; SD: standard deviation; SC: sophocarpine; Ver: verapamil. Values are expressed as mean ± SD (*n* = 10). ^∗∗∗^*P* < 0.001, compared with the NS control group.

Compared with NS, treatment with SC (80 mg/kg) effectively prolonged the postlicking foot latency (*P* < 0.001). Compared with the administration of SC alone, 80 mg/kg SC+0.3 mg/kg verapamil significantly enhanced the hot plate pain threshold of mice (*P* < 0.001). In contrast, treatment with 80 mg/kg SC+5 mg/kg CaCl_2_ reduced the threshold value; this effect was antagonized by CaCl_2_ (Figure 4).

### 3.8. Xylene-Induced Ear Oedema

Treatment with dexamethasone (2.5 mg/kg) significantly decreased the xylene-induced mouse ear oedema compared with the NS (*P* < 0.001). In this test, the administration of SC showed a dose-dependent edema-inhibiting effect (*P* < 0.01; *P* < 0.001). At 20 mg/kg, SC caused an inhibition of 17.9%. The greatest inhibition of edema was exhibited at the higher dose of 80 mg/kg SC (59.95%); this value was closer to that obtained for dexamethasone (73.53%; Table 4).

### 3.9. Acetic Acid-Induced Vascular Permeability in Mice

Treatment with dexamethasone (2.5 mg/kg) inhibited acetic acid-induced dye extrusion into the peritoneal cavity by 60.1%. The administration of SC exerted a dose-dependent inhibitory effect on dye extrusion. At 80 mg/kg, the inhibition rate induced by SC was 45.4% (Table 5).

### 3.10. Expression of IL-1*β*, IL-6, and PGE2

**Figure 5:**
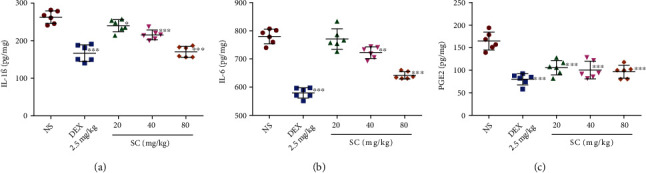
Effects of SC on the carrageenan-induced expression of IL-1*β* (a), IL-6 (b), and PGE2 (c) in mice with inflammatory pain. Abbreviations: DEX: dexamethasone; IL-1*β*: interleukin 1*β*; IL-6: interleukin 6; NS: normal saline; PGE2: prostaglandin E2; SD: standard deviation; SC: sophocarpine. Values are expressed as mean ± SD (*n* = 6). ^∗^*P* < 0.05, ^∗∗^*P* < 0.01, ^∗∗∗^*P* < 0.001, compared with the NS control group.

Compared with NS, treatment with SC (40 or 80 mg/kg) significantly decreased the expression levels of proinflammatory factors IL-1*β*, IL-6, and PGE2 in the damaged tissues of mice (*P* < 0.01; *P* < 0.001; Figure 5).

## 4. Discussion

Male mice were used in all analgesic and anti-inflammatory models because pain sensitivity changes across the menstrual cycle [18], and estrogen exerts anti-inflammatory activity [19]. Hence, female mice were excluded from this analysis to avoid any fluctuation in the results. The heat tail-flick test was used to investigate the central analgesic activity [20], and the degree of pain was determined by observing the time when mice shook their tails away from the stimulus source due to thermal stimulation. The acetic acid-induced writhing test [21] was utilized to observe the peripheral analgesic effects. Of note, the acetic acid-induced writing test is effective but nonselective [22]. Therefore, the analgesic effect of SC was further investigated using the formalin test. Subcutaneous injection of formalin induced two distinct pain responses. The first phase is acute pain caused by direct stimulation of peripheral pain-sensitive neurons by formalin, while the second phase is chronic pain caused by activation of ventral horn neurons at the spinal cord level. The hot plate test is a well-validated model that animals were only subjected to thermal stimulation once, resulting in very slight tissue damage, and the experimental results were repeatable and reliable for the detection of the analgesic effects of several types of drugs [23]. Xylene-induced ear swelling model in mice is widely used in the evaluation of anti-inflammatory drug efficacy and anti-inflammatory activity screening of compounds. Xylene is a commonly used inflammatory agent, which can induce the release of histamine, kinin, and fibrinolytic enzyme and then cause the increase of local capillary permeability, inflammatory cell infiltration, and acute exudative inflammatory edema. The results showed that SC showed effective analgesic activity in formalin test, hot plate test, and heat tail-flick test. SC exerted a good analgesic effect. SC inhibited the infiltration of local inflammatory cells and the release of inflammatory mediators, thus produced a significant inhibitory effect on xylene-induced ear swelling inflammation in mice, caused capillary contraction, and reduced acetic acid-induced vascular permeability, which proved that SC had a good anti-inflammatory effect on acute inflammation. TNF-*α* is a major cytokines, involved in acute and chronic inflammation, inducing other cytokines, including IL-1*β* and IL-6, activating the neutrophils and upregulation adhesion molecules. TNF-*α* can also promote the synthesis of PGE2, leading to blood flow changes and the resulting edema. In order to determine the mechanism of action of SC as an anti-inflammatory agent, the levels of IL-1*β*, IL-6, and PGE2 in the supernatant of mouse toe homogenate were analyzed. The results indicated that SC significantly reduced the expression of IL-1*β*, IL-6, and PGE2 [24] in the inflammation model. Hence, the anti-inflammatory effect of SC may be related to cytokine levels.

Calcium (Ca^2+^) is involved in pain regulation. Blocking the internal flow of calcium ions can affect the sensory conduction process and produce an anti-injury response [25]. NO is a small inorganic molecule that plays an important role in signal transmission in the central and peripheral nervous system. It increases the levels of guanosine cyclophosphate in neurons, which is the basis of central sensitization mediated by protein kinase G. Activation of protein kinase G and cyclic guanosine phosphate can increase the intracellular Ca^2+^ concentration by modifying voltage-gated Ca^2+^ channels and mobilizing intracellular Ca^2+^ reservoir release. The increased intracellular Ca^2+^ concentration in turn activates calmodulin, which activates NO synthase to synthesize NO. As a second messenger, NO reversibly diffuses across the cell membrane to the presynaptic primary afferent terminal. This process further enhances the release of excitatory amino acids and neuropeptides and participates in the signal transmission of harmful reactions [26]. The analgesic effect of SC was antagonized by CaCl_2_ and enhanced by verapamil. These findings suggest that SC may affect Ca^2+^ influx, thereby reducing the production of NO and subsequently exerting analgesic effects.

## Figures and Tables

**Table 1:** Effect of SC on acetic acid-induced writhing in mice.

Group	Dose (mg/kg)	Number of writhes	Inhibition (%)
NS	—	28.38 ± 7.63	—
ASP	100	6.08 ± 3.95^∗∗∗^	78.59
SC	20	24.15 ± 7.34	14.91
	40	20.46 ± 8.70^∗^	27.91
	80	14.85 ± 6.04^∗∗∗^	47.70

Abbreviations: ASP: aspirin; NS: normal saline; SD: standard deviation; SC: sophocarpine. Values are expressed as mean ± SD (*n* = 10). ^∗^*P* < 0.05, ^∗∗∗^*P* < 0.001, compared with the NS control group.

**Table 2:** Effect of SC on the tail-flick heat response in mice.

Group	Dose (mg/kg)	Response latency (s)				
		0 h	0.5 h	1 h	1.5 h	2 h
NS	—	3.82 ± 1.43	3.88 ± 1.19	3.84 ± 1.76	3.90 ± 1.26	3.77 ± 1.66
ASP	100	3.80 ± 1.11	4.90 ± 0.86^∗^	4.30 ± 1.47	5.00 ± 1.06^∗^	4.75 ± 1.00
SC	20	3.87 ± 1.10	5.90 ± 2.51^∗^	5.05 ± 1.70	5.70 ± 2.25^∗^	4.95 ± 1.43
	40	3.89 ± 1.12	5.89 ± 2.28^∗^	5.73 ± 1.84^∗^	6.03 ± 1.55^∗∗^	5.54 ± 1.91^∗^
	80	3.83 ± 1.47	6.05 ± 2.08^∗^	6.18 ± 1.33^∗∗^	5.99 ± 2.20^∗^	5.64 ± 2.11^∗^

Abbreviations: ASP: aspirin; NS: normal saline; SD: standard deviation; SC: sophocarpine. Values are expressed as mean ± SD (*n* = 10). ^∗^*P* < 0.05, ^∗∗^*P* < 0.01, compared with the NS control group.

**Table 3:** Results of the hot plate test for the effect of SC on mice.

Group	Dose (mg/kg)	Response latency (s)					
		0 min	15 min	30 min	60 min	90 min	120 min
NS	—	19.42 ± 1.02	18.47 ± 1.95	18.36 ± 1.32	20.73 ± 1.85	20.84 ± 1.98	19.74 ± 2.16
ASP	100	21.55 ± 1.75	26.20 ± 1.55^∗∗∗^	28.56 ± 2.35^∗∗∗^	21.82 ± 1.49	17.65 ± 1.44^∗∗∗^	18.59 ± 1.76
SC	20	20.38 ± 2.15	21.01 ± 2.23^∗^	22.04 ± 3.65^∗∗^	23.28 ± 1.59^∗∗^	25.62 ± 3.20^∗∗∗^	27.98 ± 3.13^∗∗∗^
	40	22.21 ± 2.32	23.05 ± 2.09^∗∗∗^	24.99 ± 2.82^∗∗∗^	26.71 ± 2.23^∗∗∗^	23.77 ± 2.68^∗^	25.04 ± 1.66^∗∗∗^
	80	21.94 ± 2.42	25.81 ± 1.17^∗∗∗^	27.80 ± 3.12^∗∗∗^	29.48 ± 1.96^∗∗∗^	28.85 ± 2.40^∗∗∗^	26.72 ± 2.44^∗∗∗^

Abbreviations: ASP: aspirin; NS: normal saline; SD:standard deviation; SC: sophocarpine. Values are expressed as mean ± SD (*n* = 10). ^∗^*P* < 0.05, ^∗∗^*P* < 0.01, ^∗∗∗^*P* < 0.001, compared with the NS control group.

**Table 4:** Effect of SC on xylene-induced ear swelling in mice.

Group	Dose (mg/kg)	Swelling	Inhibition (%)
NS	—	33.53 ± 4.74	—
DEX	2.5	8.88 ± 3.24^∗∗∗^	73.53
SC	20	27.53 ± 3.99^∗∗^	17.90
	40	22.46 ± 2.82^∗∗∗^	33.02
	80	13.43 ± 4.51^∗∗∗^	59.95

Abbreviations: NS: normal saline; SD: standard deviation; DEX: dexamethasone; SC: sophocarpine. Values are expressed as mean ± SD (*n* = 10). ^∗∗^*P* < 0.01, ^∗∗∗^*P* < 0.001, compared with the NS control group.

**Table 5:** Effect of SC on acetic acid-induced vascular permeability in mice.

Group	Dose (mg/kg)	OD	Inhibition (%)
NS	—	0.87 ± 0.21	—
DEX	2.5	0.35 ± 0.12^∗∗∗^	60.09
SC	20	0.59 ± 0.19^∗∗^	31.70
	40	0.56 ± 0.16^∗∗^	36.13
	80	0.48 ± 0.17^∗∗∗^	45.36

Abbreviations: DEX: dexamethasone; NS: normal saline; OD: optical density; SD: standard deviation; SC: sophocarpine. Values are expressed as mean ± SD (*n* = 10). ^∗∗^*P* < 0.01, ^∗∗∗^*P* < 0.001, compared with the NS control group.

## Data Availability

The data used to support the findings of this study are included in the article.
